# Evaluating deep learning approaches for AI-assisted lung ultrasound diagnosis: an international multi-center and multi-scanner study

**DOI:** 10.1186/s13089-025-00451-3

**Published:** 2025-10-06

**Authors:** Mario Muñoz, Xi Han, Jorge Camacho, Tiziano Perrone, Andrea Smargiassi, Riccardo Inchingolo, Yale Tung-Chen, Libertario Demi

**Affiliations:** 1 Institute for Physical and Information Technologies, Spanish National Research Council, Madrid, Spain; 2 Electronic Department, Universidad de Alcalá, Madrid, Spain; 3 Department of Information Engineering and Computer Science, University of Trento, Trento, Italy; 4 Medicina Interna e Medicina d’Urgenza, Humanitas Gavazzeni, Bergamo, Italia; 5 Dipartimento Neuroscienze, Organi di Senso e Torace, UOC Pneumologia, Fondazione Policlinico Universitario Agostino Gemelli IRCCS, Rome, Italy; 6 Department of Internal Medicine, Hospital Universitario La Paz, Madrid, Spain

**Keywords:** Lung ultrasound (LUS), Severity score, Artificial intelligence, Deep Learning, Assisted diagnosis, Classification, Segmentation

## Abstract

Lung ultrasound (LUS) interpretation is often subjective and operator-dependent, motivating the development of automated, artificial intelligence (AI)-based methods. This international, multi-center study evaluated two distinct deep learning approaches for automated LUS severity scoring for pulmonary infections caused by COVID-19: a pre-trained classification model (CM) and a segmentation model based method (SM); assessing performance at video, exam, and prognostic levels. Two datasets were analyzed: one comprising data from multiple scanners and another using data from a single scanner. Results showed that the SM achieved prognostic-level agreement with expert clinicians comparable to that of the CM. Furthermore, at the exam level, over 84% of examinations were classified with acceptable error (≤ 10 score difference) across both models and datasets, reaching both methods an agreement higher than 95% on the dataset acquired by a single scanner. The obtained results demonstrate the potential of AI-assisted LUS for reliable prognostic assessment and highlight that image quality and acquisition technique are key factors in achieving consistent and generalizable model performance, as well as the potential for international clinical translations.

## Introduction

Lung ultrasound (LUS) has rapidly evolved into an essential tool for assessing pulmonary conditions in a variety of clinical scenarios, particularly in critical care and emergency medicine. Its portability, safety, lack of ionizing radiation, and cost-effectiveness make it an attractive alternative to other imaging techniques, especially in resource-limited scenarios or for bedside assessments [1]. Its utility has been particularly recognized in the context of infectious diseases, such as pneumonia caused by COVID-19 infection [2, 3], where it played a crucial role in assessing lung involvement and monitoring patients by identifying characteristic sonographic features, including lung consolidations, pleural effusions, vertical artifacts and pleural irregularities [4–6].

However, despite its advantages, the accurate interpretation of LUS images requires significant expertise and experience. Identifying and classifying sonographic patterns can be challenging, even for trained clinicians, and is susceptible to inter-observer variability [7–9]. This subjectivity can impact diagnostic accuracy and patient management, highlighting the need for tools that can assist clinicians in LUS interpretation [10].

Artificial intelligence (AI) has emerged as a promising solution to further reduce the inter-observer variability and enhance the diagnostic capabilities of LUS, as well as in other medical fields [11, 12]. AI algorithms, particularly deep learning models, excel at recognizing complex patterns and features in signals and medical images, enabling enhanced analysis and interpretation. For example, in the field of radiology, deep learning models have been successfully applied to detect and classify various abnormalities in chest X-rays, such as pneumonia, pneumothorax, and lung nodules [13, 14]. In cardiology, AI algorithms have been proposed to analyse electrocardiograms (ECGs) for the early detection of heart arrhythmias and other cardiovascular diseases [15].

In the context of LUS, AI models can be broadly categorized into two types: segmentation models, which focus on delineating characteristic ultrasound artifacts (e.g., vertical artifacts, pleural line irregularities, or areas of consolidation), and classification models, which aim to categorize LUS images into predefined classes (e.g., normal vs. abnormal tissue or severity scores).

Several studies have shown promising results in applying AI to LUS interpretation. For instance, in [16] a deep learning model was developed for the real-time multi-class segmentation of artifacts, achieving high accuracy and speed. In [17], Deep Learning (DL) was applied to lung ultrasound videos for scoring pneumonia in COVID-19 patients, demonstrating the potential of AI for the assessment of lung abnormalities. Furthermore, in [18, 19] DL models were presented for the detection and localization of vertical artifacts and COVID-19 markers in LUS images, respectively, demonstrating the potential of AI for various clinical applications.

One of the scoring systems for LUS severity classification is presented in [5] ranging from 0 to 3, where:

- 0 indicates an aerated lung appearance. The pleura line is continuous and horizontal artifacts (A-lines) are present,
- 1 represents mild abnormalities, vertical artifacts without broken pleural line are visible,
- 2 corresponds to moderate abnormalities. The pleural line is broken with vertical artifacts affecting < 50% of the pleura. Small to large consolidated areas could appear.
- 3 indicates severe abnormalities, where wide vertical artifacts appear affecting > 50% of the pleural line with or without extensive consolidations.

**Fig. 1:**
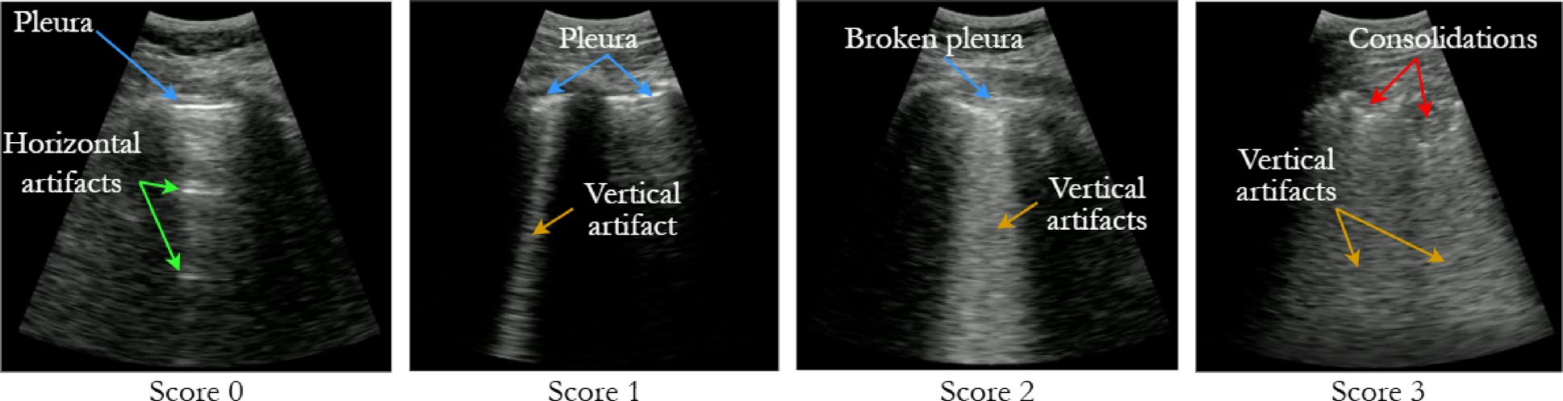
LUS score classification

In Fig. 1, an example of the LUS score image classification is shown. These individual scores are then combined to generate an overall lung score, which provides an objective and standardized assessment of lung involvement, aiding in diagnosis, prognosis, and treatment decisions [20, 21]. However, the accurate assignment of these scores to LUS videos can be challenging, as it often relies on subjective visual assessment and the determination of a threshold for the presence and extent of lung abnormalities. Furthermore, clinicians need to decide how many frames with a particular abnormality are required to assign a specific score to the entire video. Currently, this threshold is often determined subjectively, leading to potential inconsistencies in scoring.

While the literature demonstrates the potential of DL in LUS, a question remains regarding the generalizability and performance of different AI paradigms. To date, no large-scale study has performed a direct evaluation of DL-based methods across international, multi-center, and multi-scanner LUS datasets. This gap in the literature makes it difficult for the clinical and scientific community to assess the robustness of these distinct approaches in real-world settings, where data heterogeneity caused by patient population differences, acquisition protocols, and scanner variations represents a major challenge.

Building on our previous work in deep learning-based algorithms for real-time LUS assisted diagnosis [22] and AI-based scoring systems for assessing lung abnormality severity [23], this study aims to address this gap by evaluating the performance of different computational approaches in LUS scoring on an international multi-center, multi-scanner dataset. The goal is to demonstrate how these approaches can assist clinicians in LUS prognosis. The analysis is conducted at three levels: video (assessing individual LUS clips), examination (aggregating findings from all videos of a single patient to determine an overall severity score) and prognostic (predicting patient outcomes based on the LUS findings). Additionally, this research investigates the impact of scanner variability on models performance, by comparing the results obtained on videos acquired with different ultrasound scanners.

## Materials and methods

This international multi-center and multi-scanner study was conducted as a collaborative effort between the Ultrasonic Systems and Technologies Group (GSTU) at the Spanish National Research Council (CSIC) in Madrid, Spain, and the ULTRa Lab group at the University of Trento in Trento, Italy. The study involved the evaluation of two AI methods on a diverse dataset of LUS images and videos acquired from multiple centers. The details of the study design, data acquisition, AI models, and evaluation methods are described in the following subsections.

### Dataset

**Fig. 2:**
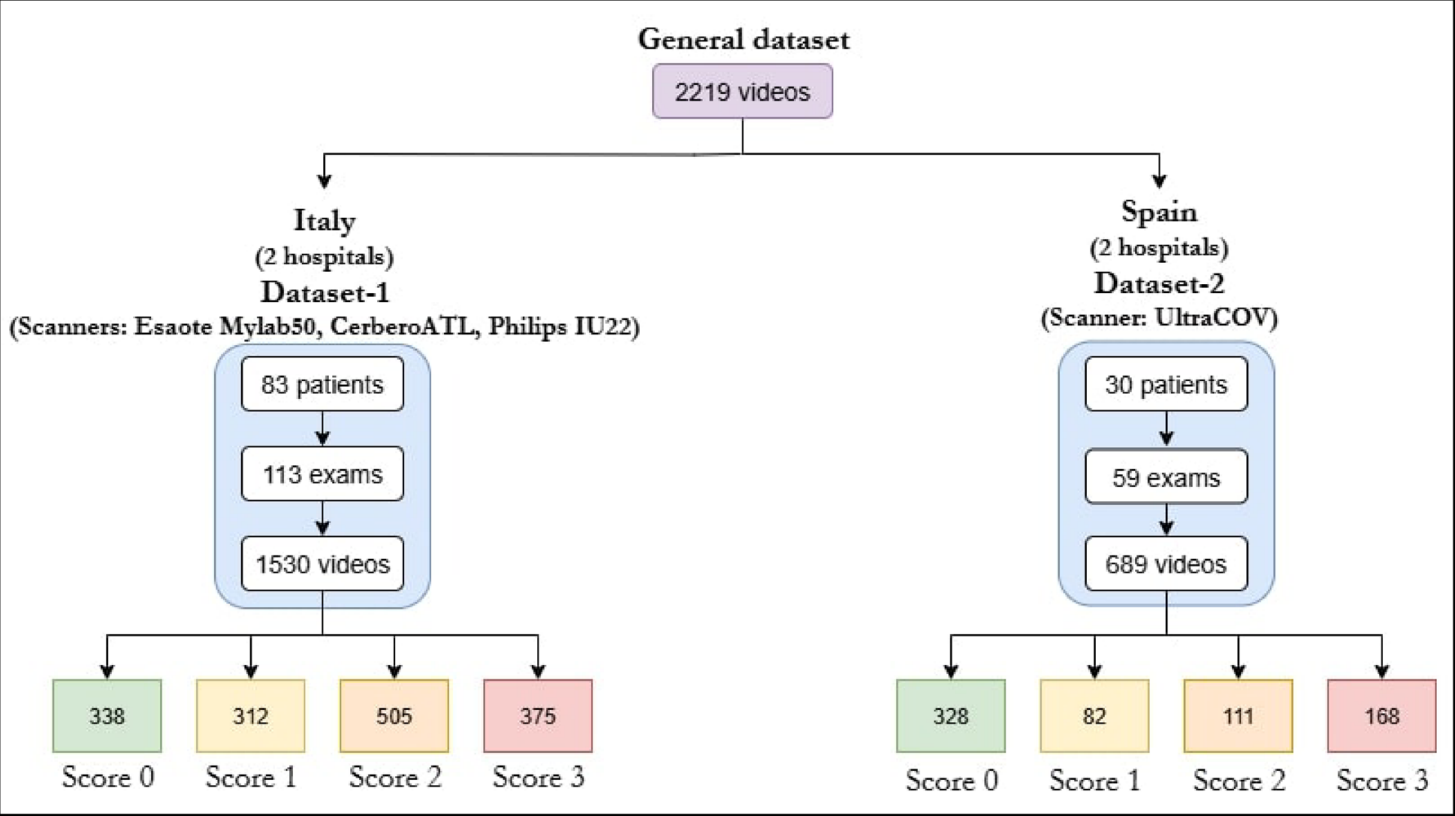
Overview of the dataset of the study

The data used to perform this study contains a total of 2219 videos consisting of 365,506 frames acquired from different hospitals in Italy and Spain of COVID-19 patients and annotated, from score 0 to score 3 on the video level, by a panel of expert clinicians, each with more than ten years of dedicated experience in thoracic ultrasound. For clarity, we will differentiate between two distinct datasets explained in detail bellow. In Fig. 2a schematic overview of the dataset is shown. Both datasets were acquired in accordance with the guidelines of the Declaration of Helsinki and approved by the Ethical Committee of the Fondazione Policlinico Universitario San Matteo (protocol 20200063198), of the Fondazione Policlinico Universitario Agostino Gemelli, Istituto di Ricovero e Cura a Carattere Scientifico (protocol 0015884/20 ID 3117) and approved by the Institutional Review Board of Hospital Universitario Puerta de Hierro (approval code PI47-21, protocol version 3.0 and date of approval 5 April 2021).

#### Dataset-1

This dataset was acquired between 2020 and 2021 in the study described in [24] and used by the ULTRa Lab team for previous research evaluating the performance of Deep Learning models in LUS prognostic. For the development of this work we will use 1530 videos acquired from 83 patients following the 14 regions’ acquisition protocol obtaining a total of 113 examinations. This multicenter dataset is composed of images from three different ultrasound scanners: Esaote Mylab50, Philips IU22 and ATL Cerbero; applying different imaging configurations: frequency from 2.5 MHz to 10 MHz and depths from 5 to 30 cm, depending on the patient and the scanner used, as explained in [24], using both convex and linear probes, reflecting a real-world multi-center and multi-scanner nature. For complete details on patient recruitment, including inclusion and exclusion criteria, readers are referred to the original publication [24].

#### Dataset-2

This dataset comprises data collected in 2021 from patients hospitalized with COVID-19. It was obtained in the clinical study described in [25] and used in previous work by the CSIC team to develop and evaluate AI algorithms for computer-aided diagnosis in LUS. The full recruitment protocol is available in the original publication [25]. It is composed of 689 LUS videos from 30 patients applying the 12 lung regions’ protocol. The UltraCOV equipment was used with a 3.5 MHz convex probe and following a standardized scanning criteria [26] where imaging configuration (focus, range, sector scan, …) and probe position was fixed trying to minimize the impact of its variability in the initial study. Each patient was examined with two standardized probe orientations: longitudinal, where the probe is aligned parallel to the ribs with its marker pointing towards the patient’s head, and transversal, where the probe is rotated 90 degrees perpendicular to the ribs. This resulted in a total of 59 examinations.

### AI methods

As previously mentioned, this study evaluates two distinct Deep Learning-based methods for computer-aided diagnosis in lung ultrasound: a classification model and a method based on segmentation models of lung ultrasound images.

#### Classification model method (CM)

The classification model utilizes a ResNet18 [27] architecture to classify lung ultrasound images according to the 4-level severity score. ResNet18 is a convolutional neural network (CNN) known for its effectiveness in image classification tasks and its ability to handle complex patterns. This model was previously developed and tested in the study described in [23], where it demonstrated good performance in classifying LUS images. The model was trained on a dataset of 58,924 LUS images acquired with a variety of scanners, including MindrayDC-70 Exp^®^, EsaoteMyLabAlpha^®^, ToshibaAplio XV^®^, and CerberoATL, as detailed in [23], including images from patients with varying degrees of lung severity [5]. As described in their methodology [23], a pre-processing step involving image cropping was applied to remove noise to the input of the network. This process, however, does not normalize the geometric aspect ratio of the underlying sonographic image.

#### Segmentation model method (SM)

**Fig. 3:**
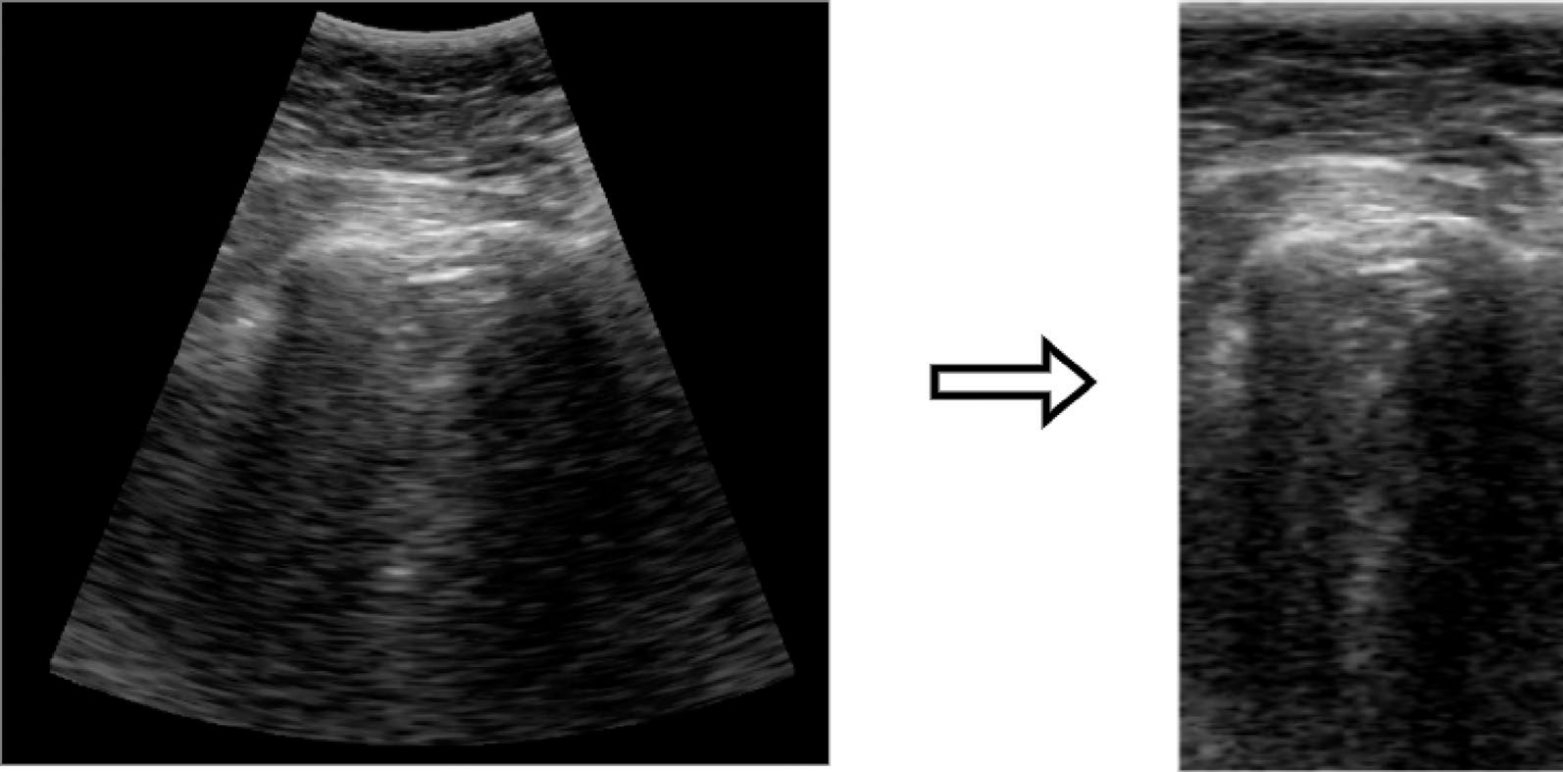
Illustration of the scan conversion pre-processing step applied in the SM method. The raw sectorial LUS image (left) is transformed into a standardized rectangular B-scan image (right) before being input into the segmentation network

The segmentation model employs an Attention U-Net architecture to segment artifacts in LUS images. Prior to being fed into the network, each raw sectorial ultrasound frame is converted into a rectangular B-scan image through a scan conversion process (see Fig. 3). This pre-processing step standardizes the input geometry, making the model independent of variations in probe sector width and shape across different scanners. The model used in this study was trained on a dataset of 9159 LUS images, acquired exclusively with the UltraCOV equipment, and its output is further processed by a post-processing algorithm described in [22], which refines the segmentation results and reduces false positives. Standard regularization techniques, including dropout, were employed during training to mitigate the risk of overfitting. This approach has shown promising results in previous studies demonstrating its ability to segment key artifacts in LUS images. Once the segmentation is performed, the presence and magnitude of different abnormalities, such as vertical artifacts and consolidations, are quantified, the severity score is assigned to each image according to the 4-level scoring system described in the introduction. It is important to note that the training set for this model included images from 27 of the 30 patients who also constitute Dataset-2.

A key advantage of this segmentation approach is its ability to provide detailed information about the location and extent of different abnormalities in LUS images. This allows for a more comprehensive assessment of lung severity compared to classification models. However, the development of accurate segmentation models for LUS faces challenges, particularly the need for large amounts of labelled data. Frame-by-frame manual annotation of LUS videos is time-consuming and requires expertise. To overcome this limitation, in [22] a semi-automatic labelling algorithm was employed reducing significantly the manual annotation effort.

### Analysis

This study employed a multi-level analysis approach, covering video, examination, and prognostic levels. By evaluating the performance of the methods at these different levels, it provides a more complete understanding of the potential benefits and limitations of AI-assisted LUS interpretation. Additionally, the performance of each method will be analyzed for each scanner at video-level, allowing for a detailed assessment of the impact of scanner variability on the results, as well as a coherence analysis between both CM and SM method.

#### Video-level analysis

At the video level, the performance of both methods was evaluated by comparing the AI-generated predictions with the ground truth annotations provided by expert clinicians. The AI models predict a severity score for each frame in a LUS video. To obtain an overall score for the video, a thresholding technique [17] was employed, by identifying the highest severity score present in a percentage of the video frames. To account for potential inter-observer variability in the ground truth annotations, the analysis was also performed with a tolerance of 1 and 2 errors per video. This means that a video was considered correctly classified if the AI prediction was within 1 or 2 score levels of the ground truth annotation, respectively.

The performance of each method at the video level was evaluated using several metrics, including:

- Accuracy: The proportion of correctly classified videos.
- F1-score: The harmonic mean of precision and recall.
- Quadratic Weighted Cohen’s Kappa (*K*_*qwc*_): A measure of agreement between two raters, taking into account chance agreement [28]. In Table 1 the interpretation of *K*_*qwc*_ is shown [29].
- Spearman’s rank correlation coefficient (ρ): A non-parametric measure used to assess the degree of correlation without assuming a linear relationship between the predicted and ground truth scores.

In addition to these metrics, confusion matrices were generated to provide a more detailed visualization of the performance of each method, showing the distribution across the different scoring classes.

**Table 1:** Table of interpretation agreement for quadratic weighted cohen’s kappa

Value	Agreement
*K*_*qwc*_ $\:\le\:$ *0*	Poor
*0 < K*_*qwc*_ $\:\le\:$ *0.2*	Slight
*0.2 < K*_*qwc*_ $\:\le\:$ *0.4*	Fair
*0.4 < K*_*qwc*_ $\:\le\:$ *0.6*	Moderate
*0.6 < K*_*qwc*_ $\:\le\:\:$*0.8*	Substantial
*0.8 < K*_*qwc*_ $\:\le\:$ *1*	Almost perfect

#### Examination-level analysis

The examination-level analysis evaluates the performance of the AI models in predicting patient-level outcomes based on the aggregation of video-level scores. For each examination, the AI-generated scores for individual LUS videos were summed to obtain an overall examination score, considering the number of lung regions explored, 12 or 14 regions, obtaining values from 0 to 36 or 42 respectively. The AI-predicted scores were then compared to the ground truth assigned by expert clinicians.

We adopted the error tolerance defined in a foundational multi-center study by Mento et al. [17], where scoring errors of ≤ 10 at the examination level were deemed clinically acceptable.

#### Prognostic-level analysis

The prognostic-level analysis aims to evaluate the ability of the AI models to predict patient outcomes based on their LUS scores. Two different prognostic classification schemes were used, depending on the LUS acquisition protocol to maintain consistency with previous studies:

- *Binary classification for 14-region protocol*: For Dataset-1, which followed a 14-region acquisition protocol, a binary classification scheme was used to categorize patients into two risk groups: low risk (score ≤ 24) and high risk (score > 24). This approach is based on the study [30], which demonstrated the ability of a 14-region LUS protocol to predict worsening in patients with COVID-19 pneumonia. Dataset-1 contains 64 exams classified by clinicians as low risk and 49 exams as high risk.
- *Multi-class classification for 12-region protocol*: For Dataset-2, which followed a 12-region acquisition protocol, a multi-class classification scheme was used to categorize patients into four severity levels: healthy (score = 0), mild (score 1–7), moderate (score 8–18), and severe (score ≥ 19). This classification is based on the study [31], which investigated the use of bedside ultrasound for the noninvasive assessment of lung lesions in patients with COVID-19. Dataset-2 contains 3 exams classified as healthy, 12 as mild, 29 as moderate and 15 as severe.

Due to these different classification schemes (binary vs. four-level), Weighted Cohen’s Kappa (*K*_*qwc*_) values are not directly comparable between Dataset-1 and Dataset-2 at the prognostic level. Therefore, while *K*_*qwc*_ is used to assess agreement within each dataset, results are presented and discussed separately.

#### AI methods agreement: CM vs. SM

To assess the agreement of the two AI methods (CM and SM), their predictions were compared at each level of analysis: video, examination, and prognostic. The agreement between the models was assessed using Weighted Cohen’s Kappa coefficient (*K*_*qwc*_), and the correlation between two models was evaluated using Spearman’s rank correlation coefficient (ρ), as well as obtaining the accuracy and F1-Score to provide additional context. This analysis allows us to evaluate the consistency of the two AI methods in interpreting LUS images and predicting patient outcomes, and to identify potential areas where the models complement each other.

## Results

### Threshold selection

**Fig. 4:**
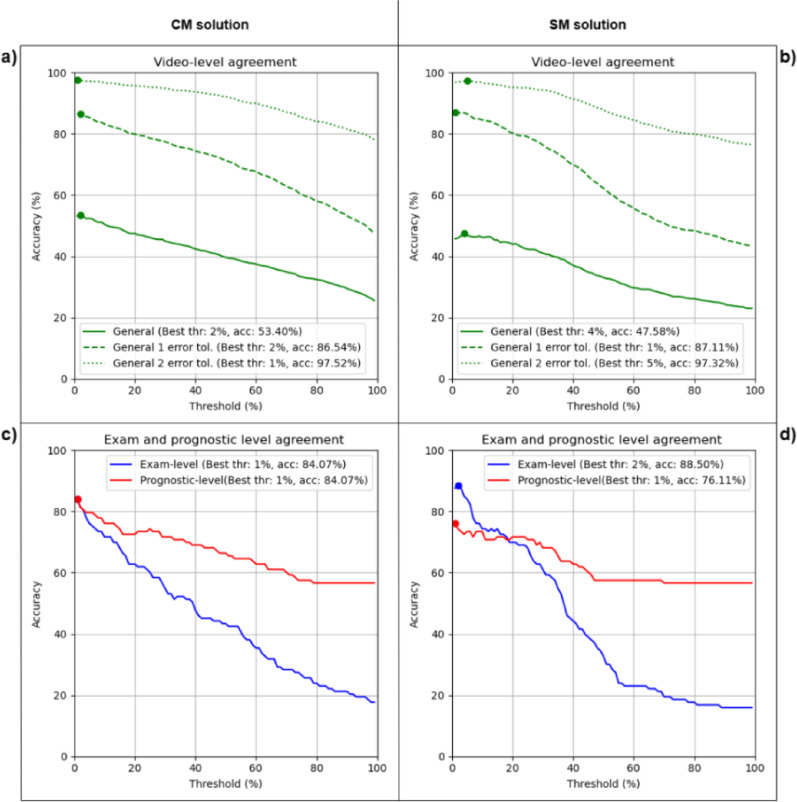
Threshold evaluation at video, examination and prognostic level for Dataset-1. **a**) and **b**) show video level performance for CM and SM respectively, while **c**) and **d**) show exam (blue) and prognostic (red) results. For each curve, the optimal threshold and its corresponding accuracy are indicated in the legend and visually marked with a point on the graph

**Fig. 5:**
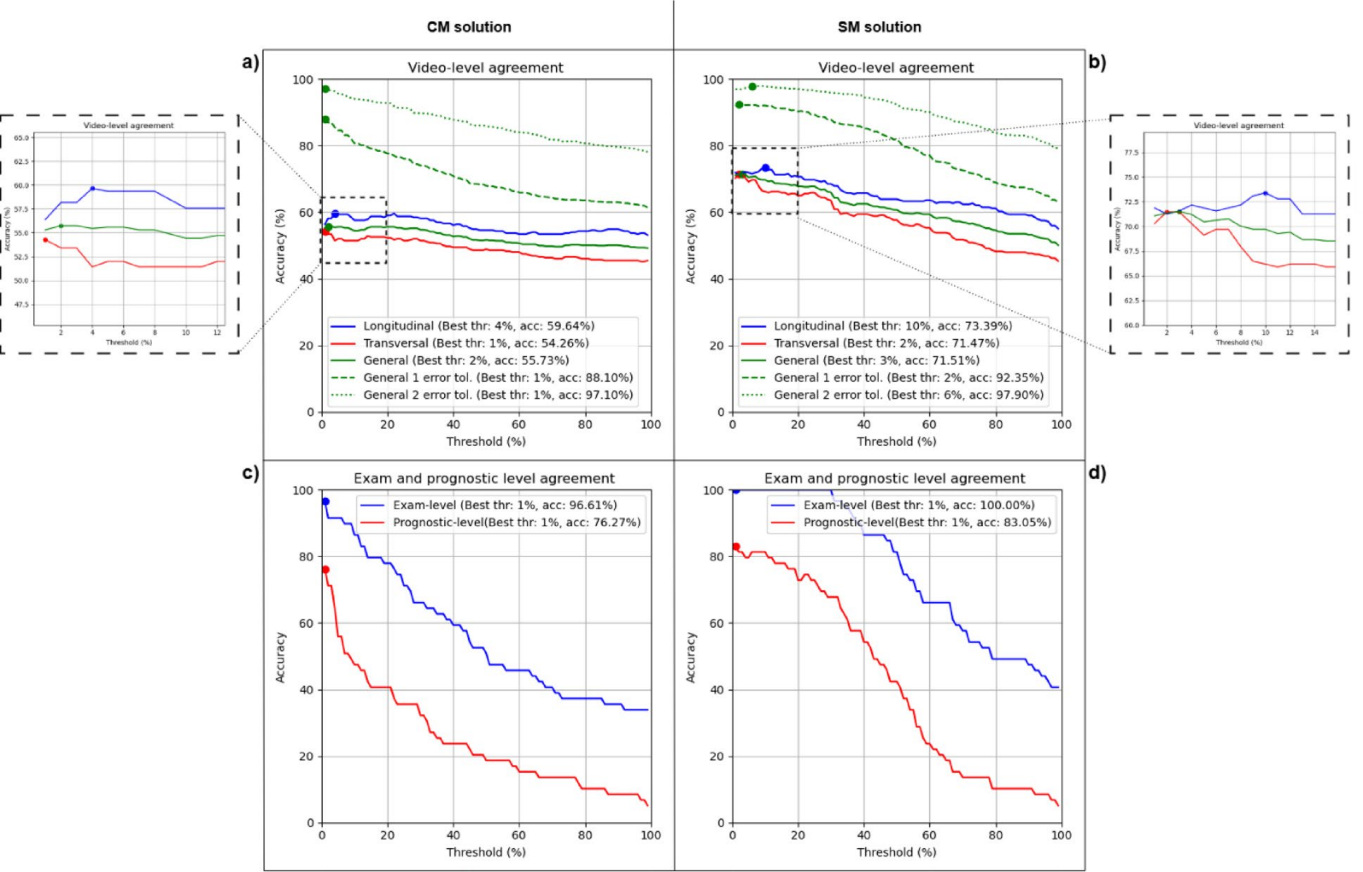
Threshold evaluation at video, examination and prognostic level for Dataset-2. **a** and **b** show video level performance for CM and SM respectively, while **c** and **d** show exam (blue) and prognostic (red) results. For each curve, the optimal threshold and its corresponding accuracy are indicated in the legend and visually marked with a point on the graph

Figures 4 and 5 show the performance of the CM and SM methods at the video, examination, and prognostic levels for different threshold values in Dataset-1 and Dataset-2, respectively. The x-axis represents the threshold and the y-axis represents the accuracy, defined as the proportion of correctly classified videos or examinations. In both cases, a range of thresholds from 1 to 100% of the frames was tested. Table 2 summarize the results on both datasets.

#### Dataset-1

In Fig. 4, we can observe the trend, in term of accuracy, of both methods. The CM method (Fig. 4a) achieves its highest agreement using a threshold of 2% obtaining an accuracy of 53.40%. On the other hand, the SM method (Fig. 4b) obtains its best result with a threshold of 4% achieving an accuracy of 47.58%. These results at video level are shown by the solid green lines in Fig. 4a and b. The dashed and dotted green lines in Fig. 4a and b represent the performance with a tolerance of ± 1 and ± 2 error respectively, where both methods show similar performance.

Figure 4c and d show the performance at the examination and prognostic levels. The red lines represent the prognostic-level accuracy, while the blue lines represent the examination-level accuracy. The CM method (Fig. 4c) achieves its best result at both levels with a 1% threshold obtaining an accuracy of 84.07%. On the other hand, the SM method (Fig. 4d) shows a higher performance at the examination level with a 2% threshold achieving an accuracy of 88.50%. However, at the prognostic level, the SM method achieves its best result with a threshold of 1% obtaining an accuracy of 76.11%.

#### Dataset-2

Figure 5 shows the performance of both solutions on Dataset-2. At the video level, the CM solution (Fig. 5a) achieves its best result with a threshold of 2% obtaining an accuracy of 55.73% without error tolerance. The SM solution (Fig. 5b) achieves its best result with a threshold of 3% obtaining an accuracy of 71.51%. At the exam level, the CM solution (Fig. 5c) maintains a good performance with a 1% threshold, obtaining an accuracy of 96.61%, while the SM solution (Fig. 5d) achieves its best result with a threshold of 1% with an accuracy of 100%. At the prognostic level, the SM solution performs better, obtaining an accuracy of 83.05% with a threshold of 1%. On the other hand, the CM solution achieves a performance of 76.27% with a threshold of 1%. Both solutions show high accuracies applying error tolerance of ± 1 and ± 2: 88.10% and 97.10% respectively for CM, and 92.35% and 97.90% for SM.

Furthermore, Fig. 5a and b also show the performance of both solutions for longitudinal (blue) and transversal (red) acquisitions separately. The results indicate that both solutions achieve similar performance for both types of acquisitions, with a slight advantage for longitudinal acquisitions.

**Table 2:** Summary of agreement with best threshold

Level	Dataset-1				Dataset-2			
	CM		SM		CM		SM	
	Acc (%)	Thr (%)	Acc (%)	Thr (%)	Acc (%)	Thr (%)	Acc (%)	Thr (%)
Video	53.40	2	47.58	4	55.73	2	71.51	3
Exam	84.07	1	88.50	2	96.61	1	100	1
Prognostic	84.07	1	76.11	1	76.27	1	83.05	1

Based on these results, a 1% threshold was selected for all subsequent video-level, examination-level, and prognostic-level analyses presented in this study.

### Video-level performance

This section examines the performance of both the Classification Model (CM) and Segmentation Model (SM) methods at the video level on Dataset-1 and Dataset-2, using the optimal 1% threshold determined in Sect. 3.1. A comparative analysis of performance across different ultrasound scanners is presented in Sect. 3.2.3. Table 3 summarizes the performance metrics for CM and SM methods on both datasets.

A detailed per-class analysis was also conducted to better understand the models’ performance on each specific LUS score. The complete precision and recall metrics for each class are presented in Appendix Table 7. These results quantitatively show that for both models, the primary source of misclassification occurred in the intermediate scores, particularly in distinguishing Score 1 from Scores 0 and 2. A full interpretation of these findings in the context of clinical challenges is provided in the Discussion section.

**Table 3:** Performance metrics for CM and SM method at video-level

Metric	Dataset-1		Dataset-2	
	CM	SM	CM	SM
Accuracy	0.53	0.46	0.55	0.71
± 1 tolerance acc	0.86	0.87	0.88	0.92
± 2 tolerance acc	0.98	0.97	0.97	0.98
F-1 Score	0.47	0.44	0.48	0.60
K_qwc_	0.63	0.58	0.66	0.79
ρ	0.65	0.59	0.64	0.80

#### CM method

The CM method showed *K*_*qwc*_ values of 0.63 (Dataset-1) and 0.66 (Dataset-2), indicating substantial agreement with the clinicians’ annotations. While overall accuracies were 0.53 and 0.55 for Dataset-1 and Dataset-2 respectively, the *K*_*qwc*_ values provide a more relevant measure of agreement beyond chance. F1-scores were 0.47 for Dataset-1 and 0.48 for Dataset-2, and Spearman’s correlation (ρ) values were 0.65 for Dataset-1 and 0.64 for Dataset-2. Accuracies with ± 1 tolerance increased to 0.86 for Dataset-1 and 0.88 for Dataset-2. With a ± 2 tolerance, accuracy further increased to 0.98 and 0.97, respectively.

**Fig. 6:**
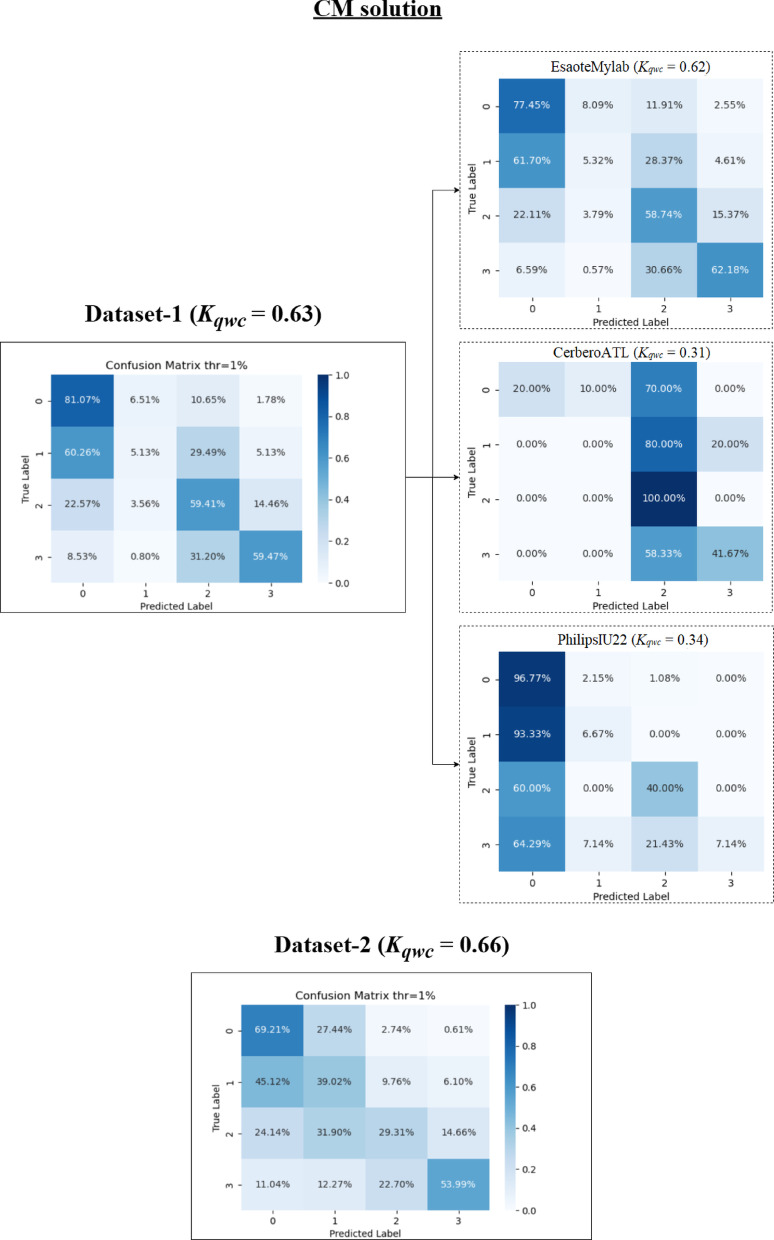
Confusion matrices for CM method in Dataset-1 and Dataset-2 as well as calculated for each machine

The overall confusion matrices for the CM method (Fig. 6) show the distribution of predicted versus true scores including the performance across different scanners, which is discussed further below. For Dataset-1, the most frequent misclassification was for videos with a true score of 1, often misclassified as 0 or 2. For Dataset-2, the confusion matrix diagonal is more consistent, corresponding to the higher *K*_*qwc*_ value.

#### SM method

The SM method achieved *K*_*qwc*_ values of 0.58 (Dataset-1, moderate agreement) and 0.79 (Dataset-2, substantial agreement). Accuracies were 0.46 and 0.71 on Dataset-1 and Dataset-2, respectively. With ± 1 tolerance, accuracies were 0.87 and 0.92. With ± 2 tolerance, accuracies were 0.97 and 0.98 respectively. F1-scores were 0.44 (Dataset-1) and 0.60 (Dataset-2), and Spearman’s ρ values were 0.59 (Dataset-1) and 0.80 (Dataset-2).

**Fig. 7:**
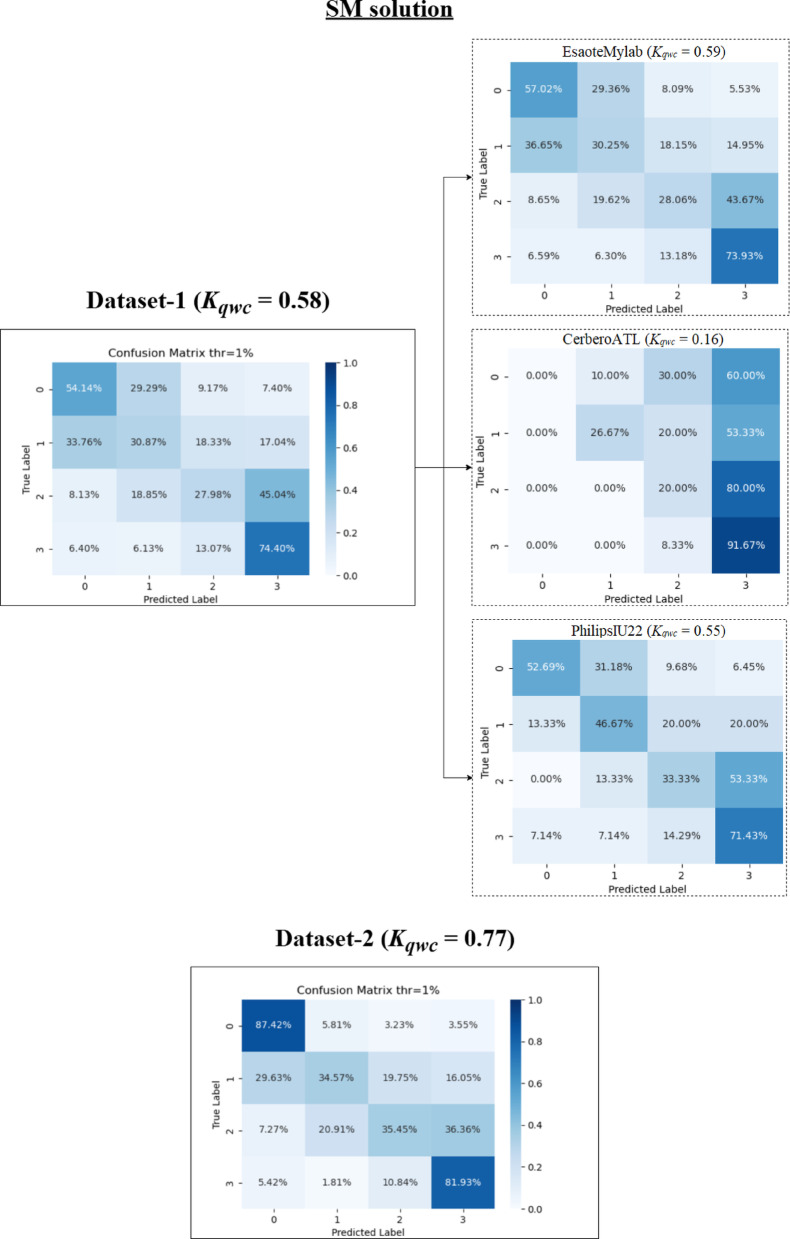
Confusion matrices for SM method in Dataset-1 and Dataset-2 as well as calculated for each machine

The overall confusion matrices for the SM method (Fig. 7) show that the model performed well classifying scores 0 and 3 in both datasets, with more misclassifications for intermediate scores in Dataset-1. The higher *K*_*qwc*_ value and stronger diagonal in the Dataset-2 confusion matrix reflect the improved performance with the standardized dataset. Figure 7 also includes results of performance for this method across different scanners, which are discussed below.

### Exam-level performance

**Fig. 8:**
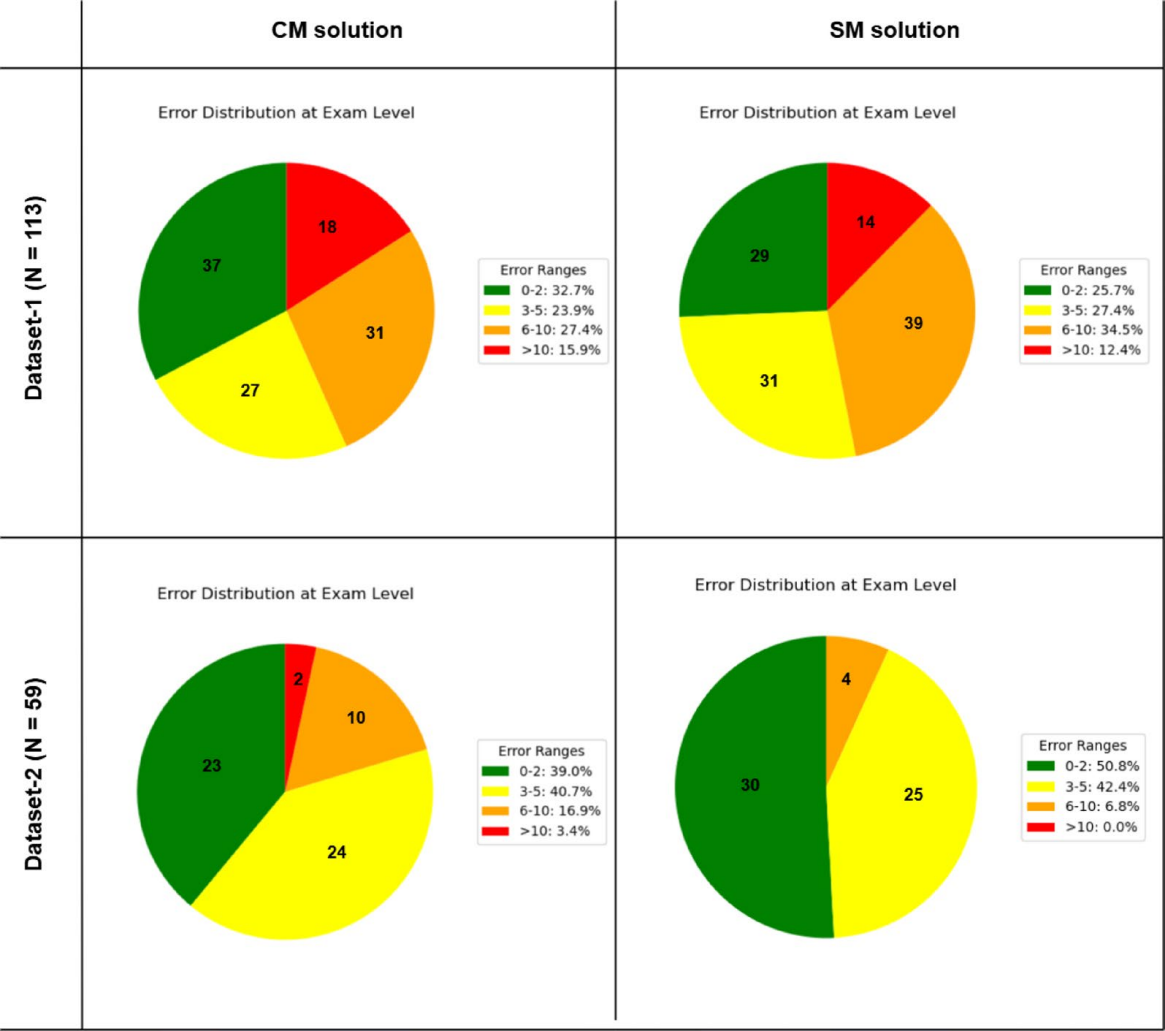
Error distribution at the examination level for CM and SM methods

This section presents the exam-level performance of the CM and SM methods, comparing AI-predicted examination scores to the ground truth scores. Figure 8 shows the distribution of scoring errors ranges between method prediction and clinician evaluation.

#### CM method

For the CM method, the error distribution differed between the two datasets (Fig. 8). 84.1% of examinations on Dataset-1 had an acceptable error (≤ 10). On Dataset-2, the CM method showed a different pattern obtaining 96.6% of examinations with an acceptable error.

#### SM method

The SM method also exhibited different error distributions across the two datasets (Fig. 8). On Dataset-1, 12.4% of exams had errors greater than 10. This resulted in 87.6% of examinations with an acceptable error on Dataset-1. On Dataset-2, the SM method showed the best performance at the examination level with no errors greater than 10, resulting in 100% of examinations with an acceptable error.

### Prognostic-level

**Fig. 9:**
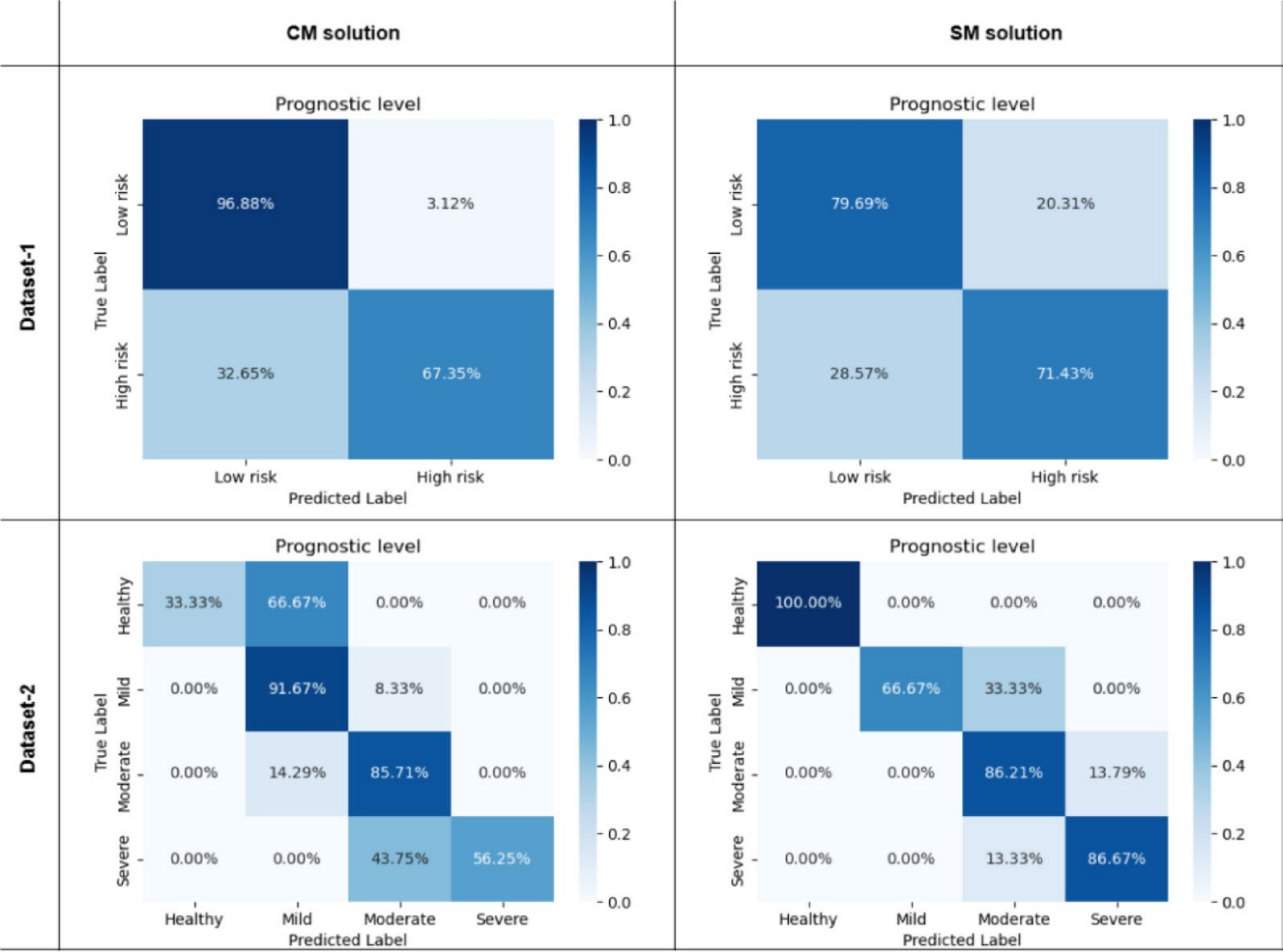
Confusion matrices at the prognostic level

This section assesses the performance of the CM and SM methods at the prognostic level. As described in the Methods section, this analysis uses different classification schemes for Dataset-1 (binary classification: low risk vs. high risk) and Dataset-2 (four-level classification: healthy, mild, moderate, severe). Performance is evaluated using F1 score, accuracy, ρ and *K*_*qwc*_, and confusion matrices which are represented in the Table 4; Fig. 9.

**Table 4:** Performance metrics for CM and SM method at prognostic level

Metric	Dataset-1		Dataset-2	
	CM	SM	CM	SM
Accuracy	0.84	0.76	0.76	0.83
F-1 Score	0.83	0.76	0.69	0.86
K_qwc_	0.66	0.51	0.80	0.87
ρ	0.69	0.51	0.82	0.84

#### CM method

For Dataset-1, the CM method achieved an accuracy of 0.84, an F1-score of 0.83, and a *K*_*qwc*_ of 0.66, indicating substantial agreement (according to Table I). The confusion matrix (Fig. 9, top left) reveals that while the model correctly classified a high percentage of ‘low risk’ cases (96.88%), it was less accurate in classifying ‘high risk’ cases (67.35% correct). On Dataset-2 the CM method achieved an accuracy of 0.76, an F1-score of 0.69, and a *K*_*qwc*_ of 0.80, also representing substantial agreement. The confusion matrix (Fig. 9, bottom left) shows moderate performance for the ‘healthy’ category (33.33%) and excellent performance for the ‘mild’ category (91.67%). However, there is more confusion between the ‘healthy’ and ‘mild’ categories, and between the ‘moderate’ and ‘severe’ categories.

#### SM method

For Dataset-1, the SM method achieved an accuracy of 0.76, an F1-score of 0.76, and a *K*_*qwc*_ of 0.51, indicating moderate agreement. The confusion matrix (Fig. 9, top right) shows that 79.69% of ‘low risk’ and 71.43% of ‘high risk’ cases were correctly classified. On Dataset-2, the SM method achieved an accuracy of 0.83, an F1-score of 0.86, and a *K*_*qwc*_ of 0.87, representing substantial agreement. The confusion matrix (Fig. 9, bottom right) reveals good performance for the ‘healthy’ (100%), ‘mild’ (66.67%), ‘moderate’ (86.21%) and ‘severe’ (86.67%) categories.

### Agreement between AI methods: CM vs. SM

This section evaluates the agreement between the predictions of both methods at the video, examination, and prognostic levels (see Table 5 for all metrics). At the video level, agreement was substantial on Dataset-1 (*K*_*qwc*_ = 0.61) and moderate on Dataset-2 (*K*_*qwc*_ = 0.52). While video-level accuracies were similar (around 0.49–0.50), the *K*_*qwc*_ values suggest a higher level of agreement beyond chance on Dataset-1. Considering tolerance for minor disagreements, the video-level accuracy with a ± 1 tolerance was 0.86 on Dataset-1 and 0.81 on Dataset-2, and with a ± 2 tolerance, it increased to 0.95 and 0.92, respectively. At the examination level, the percentage of examinations with an acceptable error (≤ 10) was 83.2% for Dataset-1 and 86.4% for Dataset-2. At the prognostic level, agreement was substantial for both Dataset-1 (*K*_*qwc*_ = 0.64, binary classification) and Dataset-2 (*K*_*qwc*_ = 0.60, four-level classification).

**Table 5:** Agreement metrics between methods: CM vs. SM

	Dataset-1				Dataset-2			
Level	Acc	F-1	K_qwc_	ρ	Acc	F-1	K_qwc_	ρ
Video	0.49	0.44	0.61	0.64	0.50	0.40	0.52	0.51
Exam	0.83	-	-	-	0.86	-	-	-
Prognostic	0.83	0.82	0.64	0.66	0.56	0.53	0.60	0.66

### Performance of each method on different scanners

Given the comparable results obtained in both datasets for both solutions, the question arises as to how they will behave on different devices and whether there may be factors affecting the implementation of AI-based methods on different devices.

For this reason, this subsection compares the performance of the CM and SM methods across the different ultrasound scanners, primarily focusing on the Weighted Cohen’s Kappa (*K*_*qwc*_) as a measure of agreement with clinician annotations. Table 6 presents the performance metrics, and Figs. 6 (CM) and 7 (SM) show the confusion matrices of method performance acquired by each scanner.

**Table 6:** Table of comparison results metrics by scanner at video-level

	Dataset-1						Dataset-2	
Metric	EsaoteMylab		CerberoATL		PhilipsIU22		UltraCOV	
	CM	SM	CM	SM	CM	SM	CM	SM
Accuracy	0.52	0.46	0.42	0.35	0.72	0.52	0.55	0.71
F-1 Score	0.46	0.44	0.34	0.27	0.39	0.43	0.48	0.60
K_qwc_	0.62	0.59	0.31	0.16	0.34	0.55	0.66	0.79
ρ	0.65	0.59	0.40	0.32	0.41	0.59	0.64	0.80

For Dataset-1, which employed EsaoteMyLab, CerberoATL, and PhilipsIU22 scanners, significant variability in agreement (*K*_*qwc*_) was observed for both the CM and SM methods.

Analyzing the CM method (Fig. 6; Table 6), the highest *K*_*qwc*_ value was achieved on the EsaoteMyLab scanner (0.62), indicating substantial agreement according to Table I. The PhilipsIU22 scanner showed a lower *K*_*qwc*_ of 0.34 (fair agreement), despite having the highest accuracy (0.72). The CerberoATL scanner exhibited the lowest *K*_*qwc*_ (0.31, fair agreement) and the lowest accuracy (0.42) for the CM method. The confusion matrix for CerberoATL reveals a tendency for the model to overestimate the severity score.

Examining the SM method (Fig. 7; Table 6), a similar pattern emerges in Dataset-1. The highest *K*_*qwc*_ was observed on the EsaoteMyLab scanner (0.59, moderate agreement). The PhilipsIU22 scanner had a *K*_*qwc*_ of 0.55 (moderate agreement), and the CerberoATL scanner had the lowest *K*_*qwc*_ (0.16, slight agreement). As with the CM method, the SM method’s confusion matrix for CerberoATL demonstrates a high degree of misclassification across all scores, except for score 3.

In contrast to Dataset-1, Dataset-2 utilized the UltraCOV scanner with standardized acquisition settings, which demonstrates consistently higher agreement for both models. The CM method achieved a *K*_*qwc*_ of 0.66 (substantial agreement), and the SM method achieved a *K*_*qwc*_ of 0.79 (substantial agreement). The confusion matrices for Dataset-2 (Figs. 6 and 7) show a clearer diagonal for both models, indicating better overall agreement with the clinician annotations.

## Discussions

The main goal of this study was to explore the potential of multi-center clinical translation by analysing LUS severity classification performance of different computational approaches across datasets from multiple institutions. We compared a classification model (CM) and a segmentation model (SM), originally developed for artifact segmentation and adapted for severity scoring, across video, examination, and prognostic levels. This comparison, performed on two datasets with varying scanner characteristics, yielded several important insights regarding model performance, generalizability, and the impact of data heterogeneity.

Before delving into the specific findings, it is important to frame the context of our performance evaluation. A single metric, such as raw accuracy, can be misleading when assessing an ordinal scoring task like LUS. For this reason, our analysis relies on a holistic interpretation of a full suite of metrics, including the confusion matrices and, most importantly, the Quadratic Weighted Cohen’s Kappa (*K*_*qwc*_). The *K*_*qwc*_ is particularly relevant as it offers a more robust measure of clinical agreement by accounting for chance and the degree of error. This is crucial given the intended role of these models as prognostic support systems, where close agreement with an expert is more valuable than predicting the exact score in every instance.

One notable finding was the comparable performance between the CM and SM methods, particularly at the prognostic level. While the SM method showed slightly higher *K*_*qwc*_ values overall, both models achieved substantial agreement with clinician annotations on both datasets at this level (CM: *K*_*qwc*_ 0.66 Dataset-1, 0.80 Dataset-2; SM: *K*_*qwc*_ 0.51 Dataset-1, 0.87 Dataset-2). This suggests that a segmentation model, despite it was initially trained for a different task (artifact segmentation), can be effectively adapted for severity scoring, achieving results comparable to a pre-trained classification model. While the CM takes into account the whole image, the SM, using the segmentation masks, can obtain a relation between the different artifacts present in the image, which is similar to the clinical scoring guideline.

**Fig. 10:**
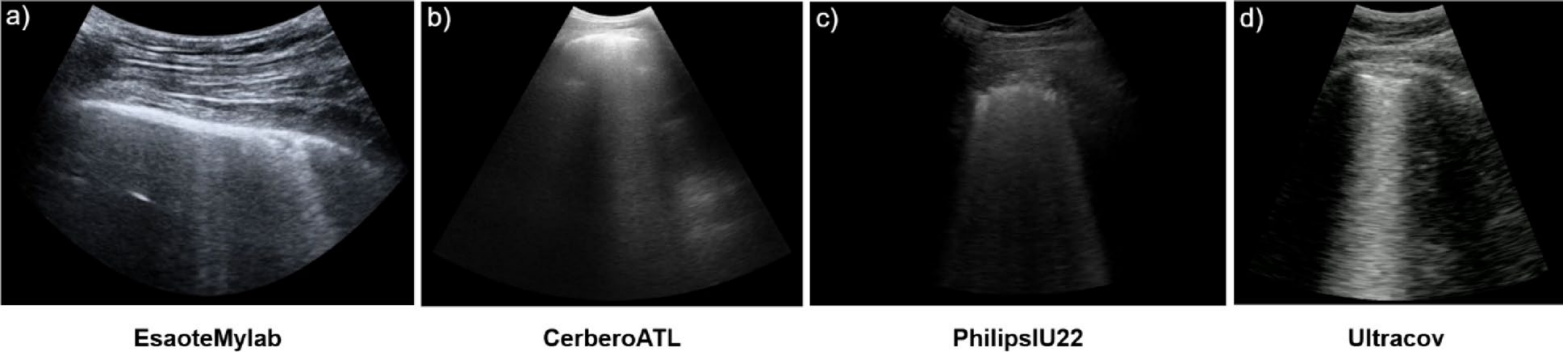
Comparison of LUS image quality across different ultrasound scanners

However, performance differences emerged at the video level, particularly concerning scanner variability. On Dataset-1 (multiple scanners), the CM and SM models exhibited significant variations in agreement with clinicians, with the CerberoATL scanner consistently yielding the lowest *K*_*qwc*_ values. Figure 10 presents representative LUS images from each of the scanners used in this study, highlighting the substantial differences in image quality. As can be seen in Fig. 10b, the image quality in ATL probes is different which is most likely the cause of the drop in performance. In contrast, the EsaoteMyLab (Fig. 10a), PhilipsIU22 (Fig. 10c), and UltraCOV (Fig. 10d) images exhibit greater clarity and detail, allowing for better visualization of artifacts and other relevant features. The confusion matrices (Figs. 6 and 7), combined with the visual differences apparent in Fig. 10, strongly suggest that these variations in image characteristics significantly impacted the models’ ability to generalize, particularly on the CerberoATL images.

In addition to the inherent differences between scanners, variations in acquisition technique also likely contributed to the observed performance differences. Factors such as probe movement and positioning, applied pressure, gain settings, and the use of pre-set imaging filters can all significantly affect image quality and the visibility of key LUS artifacts. While techniques like data augmentation and domain adaptation can help to mitigate the effects of data heterogeneity [32, 33], our findings suggest a complementary, and potentially more fundamental, approach: standardizing the image acquisition process itself. The superior performance on Dataset-2, achieved by both CM and SM despite their different training paradigms, suggest that a consistent acquisition protocol can reduce variability in image characteristics to the point where even models trained under different conditions can achieve higher accuracy and agreement. This raises the important question of whether future AI model development for LUS should prioritize training on diverse datasets from multiple scanners (to capture a wider range of variability) or on highly standardized datasets from a single or a few well-defined scanner/protocol combinations (to maximize consistency). Our results, suggest that a combination of both approaches, standardized acquisition and diverse training data, may be optimal. This is an important area for future research.

A closer inspection of the per-scanner results provides a key insight into the models’ generalization capabilities. The performance of the CM model, particularly its failure on the Philips IU22 scanner (Fig. 6), can be attributed to its sensitivity to image geometry. The CM was trained on cropped sectorial images, making it susceptible to variations in aspect ratio, which are notably different in the Philips images with their greater depth range. In contrast, the SM model’s robustness to variations in sector width stems from its pre-processing pipeline. As detailed in our Methods (Sect. 2.2.2), the conversion of all input images to a standardized rectangular B-scan format (illustrated in Fig. 3) effectively decouples the model from scanner-specific geometric properties. This geometric normalization is likely a critical factor in its ability to generalize. However, this approach is not without its own limitations; the SM’s performance can be compromised when processing images from Dataset-1 that have been cropped in depth, as this alters the aspect ratio of the resulting B-scan.

Furthermore, within the Dataset-2, a closer examination of the performance curves for longitudinal and transversal acquisitions (Fig. 5a and b) reveals a striking similarity in the trends observed for both CM and SM methods. Regardless of the method, accuracy changes in a near-parallel pattern as the threshold is varied for both longitudinal and transversal acquisitions. This suggests that, while the optimal threshold may differ slightly, the underlying relationship between the strength of the AI’s prediction (at the frame level) and the overall video-level severity score is relatively consistent across these two acquisition views. This consistency suggests that AI models can learn to exploit these features regardless of the specific probe orientation, provided that the image quality is sufficient and consistent.

The agreement between the CM and SM methods also revealed interesting patterns. While video-level agreement was higher on Dataset-1 (*K*_*qwc*_ = 0.61) than Dataset-2 (*K*_*qwc*_ = 0.52), prognostic-level agreement was substantial for both datasets (*K*_*qwc*_ = 0.64 and 0.60, respectively). This comparable inter-model agreement to the model-clinician agreement, particularly at the prognostic level, suggests that both models, despite their architectural differences, are capturing clinically relevant information. This opens the possibility of exploring ensemble methods, combining CM and SM, to potentially achieve greater robustness. This hypothesis was explored with promising results in AI-assisted CT diagnosis for COVID-19 patients in [34].

A key methodological choice in this study was the selection of the 1% threshold to translate frame-level predictions into a definitive video-level score. As shown in our results, this low threshold provided a good balance between performance at the video-level and the more clinically relevant examination and prognostic levels. This approach also ensures consistency with previous research [20] and is based on the clinical principle of focusing on the most severe findings within an ultrasound scan. However, we acknowledge this fixed threshold warrants further investigation. It may not be optimal for all videos, given the observed variability in image quality across scanners, and even within the same scanner (as suggested by the performance fluctuations in Figs. 4 and 5). A fixed, low threshold could increase sensitivity to noise or artifacts. Therefore, exploring adaptive thresholding techniques, which dynamically adjust the threshold based on image characteristics (e.g., signal-to-noise ratio) could potentially improve the robustness and accuracy of the models.

Our study has several limitations. While our sample sizes were sufficient for this initial investigation, larger datasets would be beneficial for future validation, especially to increase the statistical power of comparisons between scanners. The retrospective nature of the study is another limitation. Because we used pre-existing data, we could not control for potential selection bias in the patient population or ensure standardized LUS acquisition protocols across all patients and scanners (as highlighted by the differences between Dataset-1 and Dataset-2). This limits our ability to control for confounding sources, such as systematic differences in patient demographics (age, gender) or clinical characteristics (disease stage) between the datasets, and extract causal conclusions about the relationship between LUS findings and patient outcomes. While this data was available, a detailed cohort analysis was considered beyond the primary scope of this study, which focuses on the technical comparison of the AI models. Nevertheless, we acknowledge this as a limitation and an important avenue for future research. Furthermore, a key limitation of Dataset-2 is the limited number of healthy control examinations (total score 0). As the data acquisition focused on hospitalized COVID-19 patients, only three examinations were categorized as ‘healthy’. This small number of healthy controls makes it difficult to draw definitive conclusions about the method’s ability to accurately classify it at prognostic-level on Dataset-2, and may partially explain the observed performance differences between the CM and SM methods in classifying this specific category. Extending the study to include a larger and more representative sample of healthy individuals would be valuable for future research. Moreover, extending the study beyond COVID-19 to include patients with other pulmonary pathologies would also be valuable to assess the generalizability of the models. A potential strategy for this would be to use transfer learning to adapt the current models to new datasets containing pathologies such as bacterial pneumonia or acute heart failure. Additionally, while we used expert annotations as the ground truth, incorporating the consensus of multiple clinicians would further mitigate the inherent subjectivity of LUS interpretation.

Furthermore, a limitation related to the ground truth ambiguity is revealed by the per-class performance metrics (see Appendix Table A1). One of the most frequent sources of misclassification for both models occurred not only between Score 0 and Score 1, but also between Score 1 and Score 2. This finding should be interpreted not solely as an algorithmic limitation, but also in the context of real-world clinical challenges. The sonographic distinction between scores represents a gradual progression. Establishing a clear threshold is notoriously subjective and a well-documented source of inter-observer variability among expert clinicians [7, 8]. Therefore, the models’ difficulty in distinguishing these adjacent classes likely reflects the inherent ambiguity of the ground truth labels themselves, suggesting that the models are learning patterns consistent with human clinical interpretation challenges.

Another significant limitation to consider is that the SM method was trained on images from 27 of the 30 patients within Dataset-2. Consequently, this raises the possibility of data leakage affecting the method’s results on this dataset. While this constitutes a methodological limitation, these results are presented to ensure a comprehensive study that includes all possible model and dataset combinations.

Despite these limitations, the high prognostic-level agreement, suggests the potential clinical utility of AI-assisted LUS for risk categorization in pneumonia caused by COVID-19 infection and potentially in other respiratory diseases. This could possible improve resource allocation and patient management, increasing the number of physicians performing LUS ultrasound, and reducing inter-observer variability.

**Fig. 11:**
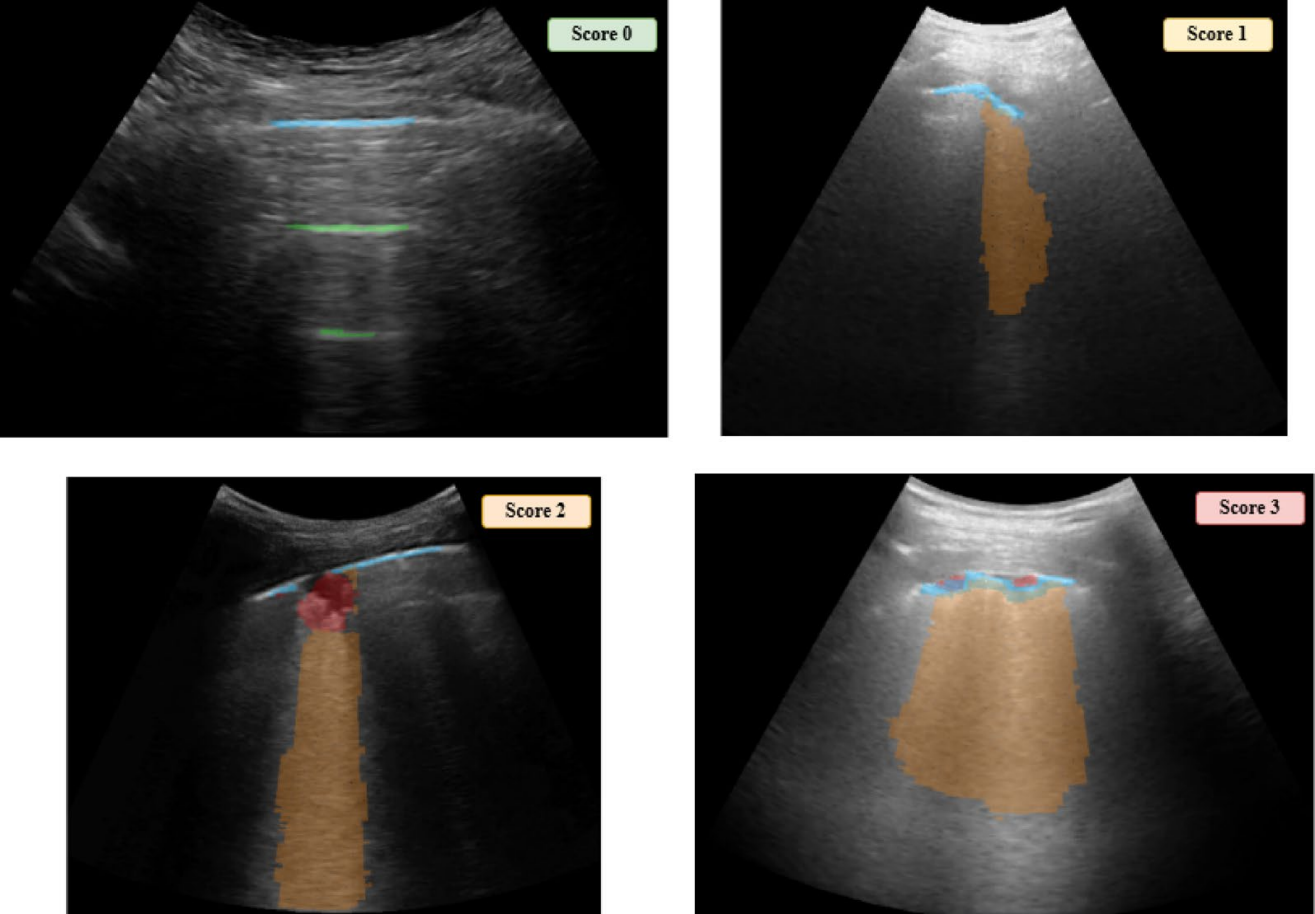
Sample of possible future implementation combining segmentation with classification on LUS: marking Pleura (blue), A-lines (green), vertical artifacts (orange), consolidations (red) and the respective predicted score. Images from Dataset-1 applying SM and CM methods

Future work should involve larger, prospective, multi-center studies with diverse patient populations and scanners. Investigating ensemble methods combining CM and SM (Fig. 11), and exploring techniques to improve model robustness to scanner variability, and promoting the standardization of LUS image acquisition protocols, are crucial.

## Conclusions

This study compared two distinct deep learning approaches, a pre-trained classification model (CM) and a segmentation model (SM) method, for automated lung ultrasound (LUS) severity assessment in patients with pulmonary infections caused by COVID-19, using datasets that reflect both multi-scanner and standardized single-scanner scenarios. It was demonstrated that AI-driven analysis of LUS has significant potential for evaluating patient risk in this context, with both models achieving accuracies over 76% comparing with expert clinicians in determining patient severity at the prognostic level (Table IV). It was also demonstrated that a segmentation model, originally designed for artifact identification, could be effectively repurposed for severity scoring, achieving a level of agreement comparable to that of a dedicated, pre-trained classification model. At the video level, while agreement with clinicians varied across datasets and models, the use of a 1% threshold on frame-level predictions proved to be a practical approach for generating video-level scores. Furthermore, the examination-level analysis highlighted that the majority of examinations across both models and datasets showed an acceptable level of error, providing further support for the feasibility of AI-assisted LUS interpretation.

Beyond demonstrating the potential for AI-powered LUS analysis, our study also revealed a critical factor influencing model performance, particularly at the prognostic level: the variability in image acquisition. The significantly improved results on Dataset-2, acquired with a single scanner and a standardized protocol, compared to the multi-scanner Dataset-1, suggest that consistent image quality, achieved through standardization, is a key determinant of reliable AI-driven prognostic assessments. This has direct implications for the successful clinical implementation of AI-assisted LUS, emphasizing the need for standardized examination protocols.

In conclusion, this research provides strong evidence for the clinical utility of AI-powered LUS analysis, particularly for prognostic assessment, while simultaneously highlighting the importance of data quality and standardization for achieving reliable and generalizable results. The similar performance between the segmentation-based method and the pre-trained classification model highlights the ability of both approaches to generalize effectively to patients and scanners excluded from the training phase. The successful completion of this study, involving the analysis of multi-center data from diverse clinical settings, underscores the crucial role of international and multidisciplinary collaborations in advancing the field of AI for LUS. Such collaborations are essential not only for accessing diverse datasets, but also for promoting the exchange of expertise necessary to develop and validate AI methods that can truly benefit patients, contributing to earlier and more accurate diagnoses.

## Data Availability

The data are available from the authors upon reasonable request.

## Appendix 1

See Table 7.

**Table 7:** Per-class precision, recall, and F1-score at the video-level

Dataset	Model	Metric	Score 0	Score 1	Score 2	Score 3
Dataset-1	CM	Precision	0.47	0.27	0.55	0.70
		Recall	0.81	0.05	0.59	0.59
		F1-Score	0.58	0.09	0.57	0.64
	SM	Precision	0.52	0.31	0.51	0.48
		Recall	0.54	0.31	0.28	0.74
		F1-Score	0.53	0.31	0.36	0.58
Dataset-2	CM	Precision	0.73	0.18	0.39	0.79
		Recall	0.69	0.39	0.29	0.54
		F1-Score	0.71	0.25	0.33	0.64
	SM	Precision	0.87	0.39	0.47	0.68
		Recall	0.87	0.35	0.35	0.82
		F1-Score	0.87	0.37	0.40	0.74
